# Adult Presentation of Subacute Necrotizing Encephalomyelopathy in Two Siblings

**DOI:** 10.7759/cureus.5522

**Published:** 2019-08-29

**Authors:** Kumail Khandwala, Fatima Mubarak

**Affiliations:** 1 Radiology, Aga Khan University Hospital, Karachi, PAK

**Keywords:** subacute necrotizing encephalopathy, leigh syndrome, magentic resonance imaging, subacute necrotizing encephalopathy, seizure disorder, spectroscopy

## Abstract

Subacute necrotizing encephalomyelopathy (SNE) or Leigh syndrome is a rare progressive neurodegenerative mitochondrial disorder typically manifesting in the pediatric age group with variable clinical presentation and genetic heterogeneity. Late-onset varieties are extremely rare and only few cases have been reported globally. Neuroimaging however shows characteristic symmetrical necrotic lesions in the basal ganglia and/or brainstem. We report cases of two siblings who had history of seizures, but presented to us in adulthood. They had similar clinical presentation and radiological features on magnetic resonance imaging (MRI) and were subsequently diagnosed with SNE primarily based on imaging.

## Introduction

Leigh syndrome, also termed subacute necrotizing encephalomyelopathy (SNE) and first described by Denis Leigh in 1951, is a rare, inherited progressive neurodegenerative disorder that usually manifests in infancy or early childhood [1]. Usual age of presentation is before six months of age in about 50% cases. Late-onset and adult-onset varieties of Leigh syndrome have been rarely described in the literature [2-4]. We report an atypical variety of SNE that presented in adulthood in two siblings who had identical clinical presentation and magnetic resonance imaging (MRI) features.

## Case presentation

An 18-year-old male presented to the emergency room (ER) with complaints of recurrent episodes of seizures for the past one week. He had a history of generalized tonic clonic seizures for the last four years and was the product of second-degree consanguineous marriage, with an uneventful perinatal history or past medical history. On initial examination, he was unconscious with Glasgow Coma Scale (GCS) of 5 and afebrile. Initial management aimed at controlling the seizures with Diazepam. His pulse was 154 beats per minute, respiratory rate 36 per minute and blood pressure 84/46 mm Hg. Central nervous system (CNS) examination showed increased tone in the lower limbs. Deep tendon reflexes were exaggerated with positive bilateral Babinski sign. Pupils were dilated and sluggishly reacting to light. Fundus examination and visual evoked potentials were normal. The above clinical findings were highly suggestive of a neurodegenerative disorder and the patient was further investigated in which cerebrospinal fluid (CSF) lactate was 3.9 mmol/L (normal <3 mmol/L) and serum lactate was 1.6 mmol/L (normal 0.5-1 mmol/L) and were found to be mildly elevated. CSF, blood and urine cultures were negative. Herpes simplex virus (HSV) by polymerase chain reaction (PCR) was negative. Mycobacterium tuberculosis (MTB) by PCR was negative. Ceruloplasmin levels of 0.29 g/L (normal 0.2-0.6 g/L) ruled out Wilson's disease. Initial electroencephalogram (EEG) showed reactive theta and delta slowing.

**Figure 1:**
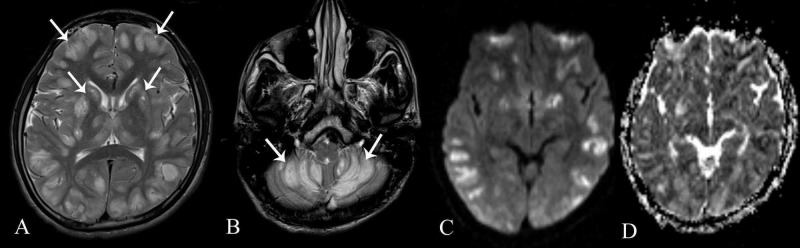
Magnetic Resonance Imaging (A & B) Axial T2-weighted images showing symmetrical abnormal T2 hyperintense signals in cortical grey matter, bilateral caudate nuclei, bilateral putamen, bilateral thalami and bilateral cerebellar hemispheres (white arrows). (C & D) Diffusion-weighted sequences and apparent diffusion coefficient maps demonstrating positive diffusion restriction in these affected areas.

MRI showed symmetrical abnormal signals in cortical grey matter, bilateral caudate nuclei, bilateral putamen, bilateral thalami and bilateral cerebellar hemispheres appearing predominantly hyperintense on T2-weighted and FLAIR images with diffusion restriction (Figure 1).

**Figure 2:**
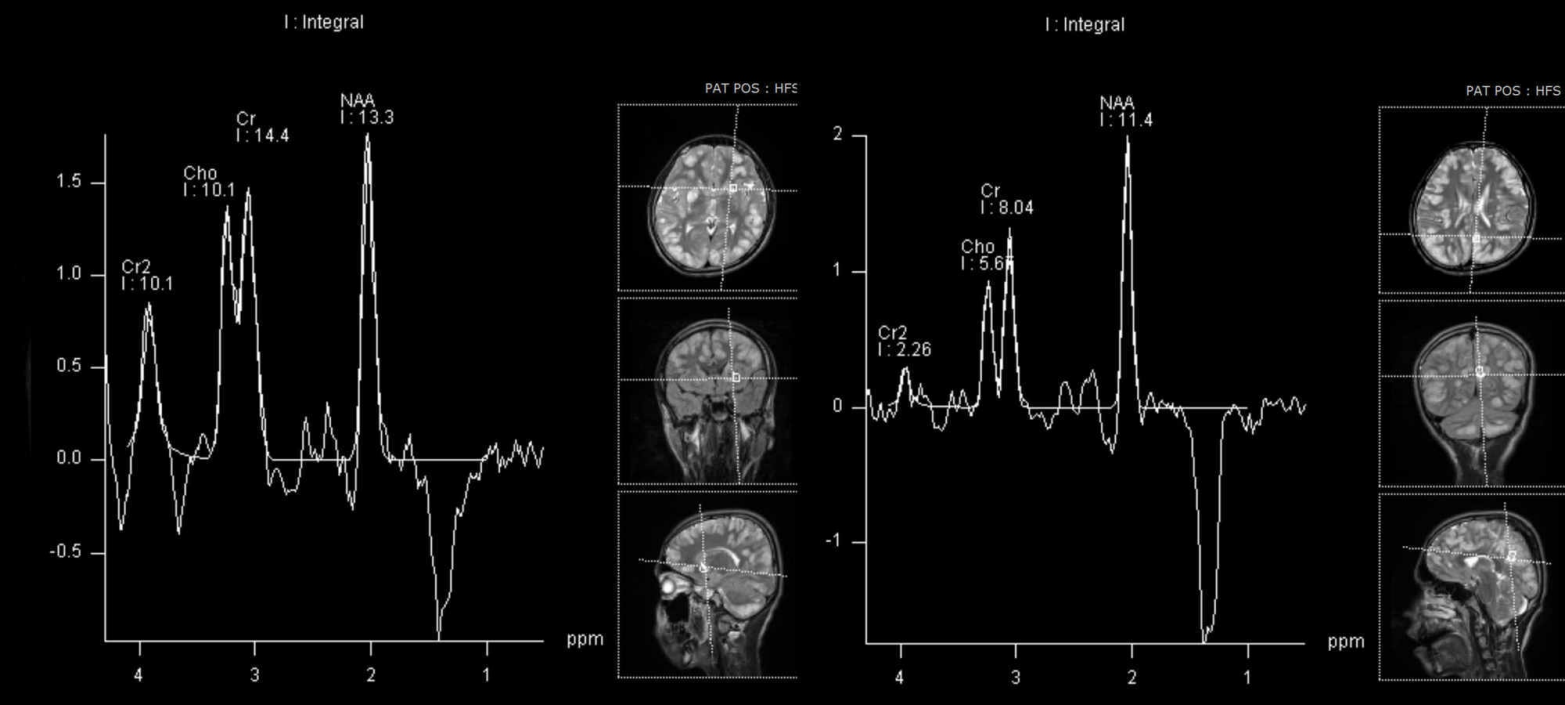
Magnetic Resonance Spectroscopy Spectroscopy done in the region of caudate nucleus and cortical matter shows inverted doublet lactate peak at 1.3 ppm.

MR spectroscopy was also done which showed inverted doublet lactate peak (Figure 2). He was managed on the lines of a suspected mitochondrial disorder and started on Co-enzyme Q-10 and L-carnitine. He was also started on Levetiracetam for symptomatic relief and was asked to follow up in the neurology clinic; however, he was subsequently lost to follow-up.

**Figure 3:**
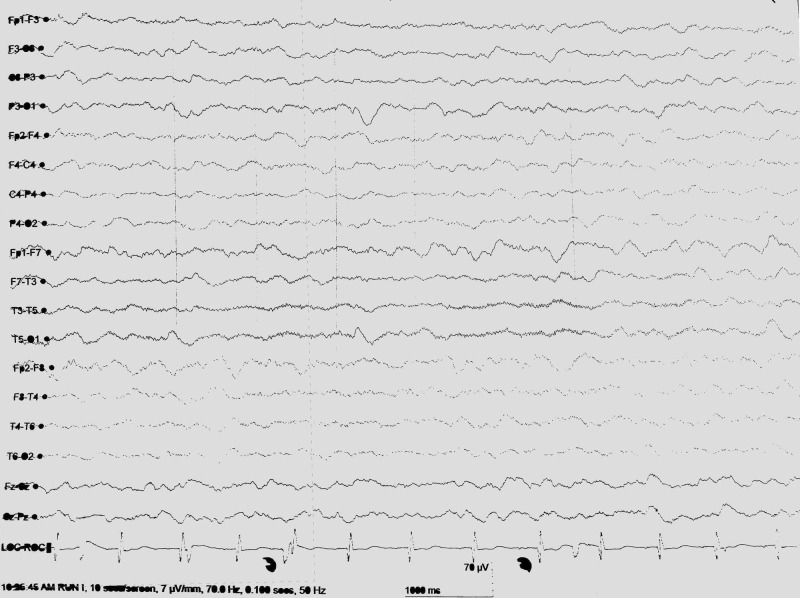
Electroencephalogram Electroencephalogram showing diffuse delta and theta wave slowing.

A few years later, his sister, a 20-year-old female, was brought to the ER with seizures and altered mentation. She also had recurrent epilepsy for five years. It was discovered on history taking that her brother who had lost to follow-up previously, died two years ago from his neurodegenerative disorder. Her GCS at the time of admission was 5/15 with hyper-reflexia, positive Babinski’s sign. Her laboratory workup showed increased creatine phosphokinase levels of 969 IU/L (normal 34-145 IU/L). CSF detailed report and culture was unremarkable. Her EEG also showed diffuse delta and theta slowing (Figure 3).

**Figure 4:**
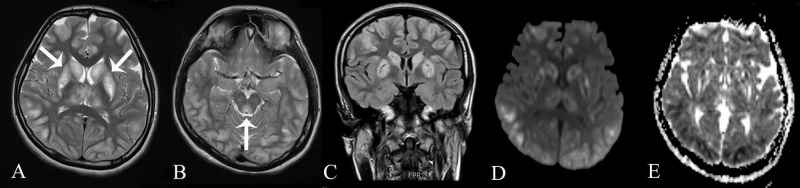
Magnetic Resonance Imaging (A) Axial T2-weighted image showing similar symmetrical T2 hyperintense lesions in cortical and deep grey matter (white arrows). (B) Axial T2-weighted image showing involvement of the periaqueductal grey (white arrow). (C) Coronal FLAIR image showing that the lesions were hyperintense on FLAIR. (D & E) Diffusion-weighted sequences and apparent diffusion coefficient maps showing patchy diffusion restriction in the affected regions.

Urine toxicology showed no evidence of amphetamines, cannabinoids, or opiates. MRI was done which showed similar hyperintense lesions in cortical and deep grey matter along with involvement of the periaqueductal grey matter. Diffusion restriction was present as well (Figure 4). She was also started on Levetiracetam for seizures and L-carnitine for symptomatic treatment. In view of the guarded prognosis, the family subsequently moved the patient to another facility due to financial constraints. Based on the clinical presentation and MRI features, a diagnosis of subacute necrotizing encephalomyelopathy (Leigh’s syndrome) was established in these two siblings.

## Discussion

Subacute necrotizing encephalomyelopathy is a rare, inherited progressive neurodegenerative disorder that usually manifests in infancy or early childhood [1]. Clinical presentation of SNE is variable. In most usual cases that present in infancy or childhood there is psychomotor delay or regression, brainstem and pyramidal signs. Dysfunction of the respiratory chain enzymes is responsible for the disease. Some cases display a maternal inheritance, others follow an autosomal recessive (pyruvate carboxylase, SURF1 gene mutations with cytochrome C oxidase (COX) deficiency, adenosine triphosphate [ATP] synthase subunit 6 and complex I deficiencies) or sex-linked (pyruvate dehydrogenase E1 gene mutations) pattern of inheritance. In some cases, the genetic cause remains unknown [5].

Despite this, the neuroimaging and pathological features of affected children are identical. Characteristic focal, bilateral, and symmetric necrotic lesions associated with demyelination are noted in the brainstem, diencephalon, basal ganglia, and cerebellum. A diagnosis of SNE can be made without histopathology on the basis of clinical signs and symptoms, mode of inheritance, metabolic abnormalities such as elevated CSF lactate, and neuroimaging findings [6,7]. In some reported cases, the diagnosis was purely made on clinical presentation and MRI findings [8,9]. Adult-onset SNE is rare, and has previously been defined as patients who survived longer than 18 years [4]. In addition, the clinical manifestations may also be different than those of children with less incidence of developmental delay as seen in our cases. Previously reported cases have excluded infections, autoimmune diseases and toxins as have we in our cases [10]. Seizures are a less common clinical presentation of this disease, however, in a recent study, clinical seizures were observed in 56% of patients with SNE. All their patients were found to have slow and disorganized background neural activity on EEG as observed in both of our cases [11].

Genetic testing, histopathology or molecular studies were not performed in our patients due to financial constraints. However, with appropriate investigations, accurate diagnosis and prompt institution of adequate supportive therapy such as with thiamine (vitamin B1), biotin (vitamin B7), succinic acid and Coenzyme Q10, symptomatic amelioration can be achieved in some cases, thereby adding life to the limited years of survival in affected patients [12].

## Conclusions

The diagnosis of subacute necrotizing encephalomyelopathy should be considered in appropriate clinical and laboratory settings whenever symmetrical abnormalities are encountered on imaging predominantly in the basal ganglia and/or brainstem. However, the adult-onset variety is extremely rare and requires a high index of suspicion for diagnosis.
